# Supplementary material for: The association between chronic bullying victimization with weight status and body self-image: a cross-national study in 39 countries
Source: PeerJ. 2018 Jan 31;6:e4330. doi: 10.7717/peerj.4330 (PMC5794335; doi:10.7717/peerj.4330)
Supplement: Supplemental Information 1 [file peerj-06-4330-s001.docx]

Table S1 The associations between perceived weight status and covariates, n (%)

|  | **Underweight** | **Normal weight** | **Overweight** | **Obese** |
| --- | --- | --- | --- | --- |
| **Sex^*^** |  |  |  |  |
| Male | 8,249(9.57) | 62,311(72.31) | 12,788(14.84) | 2,824(3.28) |
| Female | 13,978(15.97) | 63,483(72.51) | 8,388(9.58) | 1,704(1.95) |
| **Age group^*^** |  |  |  |  |
| 11 | 7,716(14.73) | 36,747(70.16) | 6,545(12.50) | 1,365(2.61) |
| 13 | 7,920(13.43) | 42,404(71.88) | 7,136(12.10) | 1,531(2.60) |
| 15 | 6,591(10.57) | 46,643(74.80) | 7,495(12.02) | 1,632(2.62) |
| **Classmate support^*^** |  |  |  |  |
| Negative | 8,411(12.42) | 47,767(70.52) | 9,338(13.79) | 2,216(3.27) |
| Positive | 13,556(13.03) | 76,599(73.65) | 11,583(11.14) | 2,267(2.18) |
| **Academic achievement^*^** |  |  |  |  |
| Good | 14,764(13.18) | 81,647(72.89) | 12,981(11.59) | 2,625(2.34) |
| Average and below | 7,179(12.06) | 42,571(71.51) | 7,944(13.34) | 1,840(3.09) |
| **SES^*^** |  |  |  |  |
| Low | 1,752(14.73) | 8,419(70.80) | 1,387(11.66) | 333(2.80) |
| Medium | 7,460(12.38) | 42,991(71.37) | 8,015(13.31) | 1,770(2.94) |
| High | 12,504(12.78) | 71,775(73.35) | 11,260(11.51) | 2,311(2.36) |

^*^ Cochran-Mantel-Haenszel test, p<0.0001
