# Supplementary material for: The association between chronic bullying victimization with weight status and body self-image: a cross-national study in 39 countries
Source: PeerJ. 2018 Jan 31;6:e4330. doi: 10.7717/peerj.4330 (PMC5794335; doi:10.7717/peerj.4330)
Supplement: Supplemental Information 2 [file peerj-06-4330-s002.docx]

Table S2 The associations between perceived body self-image and covariates, n (%)

|  | **Too thin** | **Normal** | **A little bit fat** | **Too fat** |
| --- | --- | --- | --- | --- |
| **Sex^*^** |  |  |  |  |
| Male | 17,580(17.08) | 62,146(60.38) | 20,756(20.17) | 2,442(2.37) |
| Female | 13,000(12.16) | 57,591(53.86) | 31,401(29.37) | 4,932(4.61) |
| **Age group^*^** |  |  |  |  |
| 11 | 10,046(15.08) | 40,632(60.99) | 14,058(21.10) | 1,881(2.82) |
| 13 | 9,768(13.80) | 39,836(56.27) | 18,476(26.10) | 2,709(3.83) |
| 15 | 10,459(14.81) | 38,251(54.17) | 19,208(27.20) | 2,698(3.82) |
| **Classmate support^*^** |  |  |  |  |
| Negative | 12,948(15.58) | 43,036(51.77) | 23,138(27.83) | 4,011(4.82) |
| Positive | 17,191(13.88) | 74,970(60.52) | 28,446(22.96) | 3,268(2.64) |
| **Academic achievement^*^** |  |  |  |  |
| Good | 19,202(14.27) | 80,702(59.96) | 31,026(23.05) | 3,658(2.72) |
| Average and below | 10,892(15.12) | 37,149(51.56) | 20,397(28.31) | 3,617(5.02) |
| **SES^*^** |  |  |  |  |
| Low | 2,830(19.34) | 8,236(56.30) | 3,054(20.87) | 510(3.49) |
| Medium | 10,878(14.88) | 40,996(56.09) | 18,481(25.29) | 2,735(3.74) |
| High | 15,964(13.69) | 67,296(57.69) | 29,413(25.22) | 3,973(3.41) |

^*^ Cochran-Mantel-Haenszel test, p<0.0001
