# Supplementary material for: The association between chronic bullying victimization with weight status and body self-image: a cross-national study in 39 countries
Source: PeerJ. 2018 Jan 31;6:e4330. doi: 10.7717/peerj.4330 (PMC5794335; doi:10.7717/peerj.4330)
Supplement: Supplemental Information 3 [file peerj-06-4330-s003.docx]

Table S3 The associations between chronic bullying victimization and covariates, n (%)

|  | **Non-****chronic victimization** | **Chronic victimization** |
| --- | --- | --- |
| **Sex^#^** |  |  |
| Male | 86,709(87.49) | 12,399(12.51) |
| Female | 92,872(89.91) | 10,423(10.09) |
| **Age group^*^** |  |  |
| 11 | 55,764(86.75) | 8,515(13.25) |
| 13 | 59,873(87.90) | 8,239(12.10) |
| 15 | 62,354(91.39) | 5,874(8.61) |
| **Classmate support^#^** |  |  |
| Negative | 65,961(82.03) | 14,450(17.97) |
| Positive | 111,687(93.30) | 8,024(6.70) |
| **Academic achievement^#^** |  |  |
| Good | 117,524(90.17) | 12,817(9.83) |
| Average and below | 59,755(86.12) | 9,628(13.88) |
| **SES^*^** |  |  |
| Low | 10,231(84.45) | 1,884(15.55) |
| Medium | 61,755(87.95) | 8,465(12.05) |
| High | 103,589 (89.77) | 11,804(10.23) |

^#^ Chi-Square test, p<0.0001, ^*^ Cochran-Mantel-Haenszel test, p<0.0001
