# Supplementary material for: The association between chronic bullying victimization with weight status and body self-image: a cross-national study in 39 countries
Source: PeerJ. 2018 Jan 31;6:e4330. doi: 10.7717/peerj.4330 (PMC5794335; doi:10.7717/peerj.4330)
Supplement: Supplemental Information 4 [file peerj-06-4330-s004.docx]

Table S4 The interactions between weight status, body-image and victimization, OR (95%CI, p value)

|  | **Total** | **Male** | **Female** |
| --- | --- | --- | --- |
| **Perceived weight status (base=Normal)^*^** |  |  |  |
| Underweight | 1.15(1.07-1.22, p<0.0001) | 1.20(1.07-1.35, p=0.0015) | 1.11(1.03-1.20, p=0.0059) |
| Overweight/Obese | 1.23(1.10-1.38, p=0.0004) | 1.18(1.04-1.33, p=0.0096) | 1.38(1.19-1.60, p<0.0001) |
| **Perceived body-image (base=Normal)^*^** |  |  |  |
| Too thin | 1.41(1.33-1.50, p<0.0001) | 1.38(1.30-1.46, p<0.0001) | 1.49(1.35-1.64, p<0.0001) |
| A little bit fat/Too fat | 1.68(1.59-1.76, p<0.0001) | 1.80(1.67-1.95, p<0.0001) | 1.62(1.53-1.72, p<0.0001) |
| **Interactions(base=Normal* Normal) ^*^** |  |  |  |
| Underweight*Too thin | 0.93(0.84-1.03, p=0.1681) | 0.96(0.83-1.11, p=0.5375) | 0.88(0.78-1.00, p=0.0461) |
| Underweight*A little bit fat/Too fat | 1.03(0.84-1.26, p=0.7866) | 0.83(0.58-1.18, p=0.3068) | 1.12(0.91-1.38, p=0.2711) |
| Overweight/Obese*Too thin | 0.92(0.72-1.16, p=0.4781) | 0.88(0.64-1.21, p=0.4370) | 1.13(0.71-1.78, p=0.6037) |
| Overweight/ A little bit fat/Too fat | 0.99(0.87-0.12, p=0.8682) | 0.91(0.78-1.05, p=0.2048) | 0.99(0.85-1.15, p=0.8859) |

^*^ Odds ratio adjusted for sex, age group, classmate support, academic achievement, SES, GDP per capita and GINI index.
