# Supplementary material for: The association between chronic bullying victimization with weight status and body self-image: a cross-national study in 39 countries
Source: PeerJ. 2018 Jan 31;6:e4330. doi: 10.7717/peerj.4330 (PMC5794335; doi:10.7717/peerj.4330)
Supplement: Supplemental Information 5 [file peerj-06-4330-s005.docx]

Table S5 Adjusted predicted probability of weight status, Pr (95%CI, p value)

|  | **Total** | **Male** | **Female** |
| --- | --- | --- | --- |
| **Weight status** |  |  |  |
| Underweight | 0.108(0.093-0.123,p<0.0001) | 0.126(0.105-0.147,p<0.0001) | 0.093(0.080-0.105,p<0.0001) |
| Normal weight | 0.100(0.085-0.114,p<0.0001) | 0.112(0.094-0.129,p<0.0001) | 0.088(0.072-0.101,p<0.0001) |
| Overweight | 0.131(0.113-0.149,p<0.0001) | 0.139(0.119-0.159,p<0.0001) | 0.126(0.108-0.144,p<0.0001) |
| Obese | 0.166(0.143-0.190,p<0.0001) | 0.177(0.153-0.202,p<0.0001) | 0.158(0.125-0.190,p<0.0001) |
| **Changes** |  |  |  |
| Underweight vs Normal | 0.008(0.004-0.013,p<0.0001) | 0.015(0.006-0.023,p=0.0010) | 0.005(0.000-0.009,p=0.0430) |
| Overweight vs Normal | 0.032(0.025-0.038,p<0.0001) | 0.027(0.019-0.035,p<0.0001) | 0.038(0.029-0.047,p<0.0001) |
| Obese vs Normal | 0.067(0.052-0.081,p<0.0001) | 0.066(0.052-0.079,p<0.0001) | 0.070(0.043-0.097,p<0.0001) |
| Overweight vs Underweight | 0.023(0.015-0.032,p<0.0001) | 0.013(0.003-0.023,p=0.0140) | 0.033(0.022-0.045,p<0.0001) |
| Obese vs Underweight | 0.059(0.045-0.072,p<0.0001) | 0.051(0.037-0.065,p<0.0001) | 0.065(0.037-0.093,p<0.0001) |
