# Supplementary material for: The association between chronic bullying victimization with weight status and body self-image: a cross-national study in 39 countries
Source: PeerJ. 2018 Jan 31;6:e4330. doi: 10.7717/peerj.4330 (PMC5794335; doi:10.7717/peerj.4330)
Supplement: Supplemental Information 6 [file peerj-06-4330-s006.docx]

Table S6 Adjusted predicted probability of body self-image, Pr (95%CI, p value)

|  | Total | Male | Female |
| --- | --- | --- | --- |
| **Body self-image** |  |  |  |
| Too thin | 0.121(0.104-0.138,p<0.0001) | 0.135(0.115-0.155,p<0.0001) | 0.108(0.093-0.122,p<0.0001) |
| About right | 0.090(0.077-0.104,p<0.0001) | 0.104(0.088-0.120,p<0.0001) | 0.078(0.067-0.891,p<0.0001) |
| A little bit fat | 0.129(0.113-0.145,p<0.0001) | 0.151(0.133-0.170,p<0.0001) | 0.109(0.094-0.125,p<0.0001) |
| Too fat | 0.226(0.199-0.253,p<0.0001) | 0.252(0.224-0.279,p<0.0001) | 0.201(0.174-0.229,p<0.0001) |
| **Changes** |  |  |  |
| Too thin vs About right | 0.031(0.025-0.036,p<0.0001) | 0.032(0.025-0.039,p<0.0001) | 0.030(0.024-0.036,p<0.0001) |
| A little bit fat vs About right | 0.039(0.034-0.043,p<0.0001) | 0.048(0.041-0.054,p<0.0001) | 0.032(0.026-0.038,p<0.0001) |
| Too fat vs About right | 0.136(0.118-0.154,p<0.0001) | 0.148(0.128-0.168,p<0.0001) | 0.123(0.103-0.143,p<0.0001) |
| A little bit fat vs Too thin | 0.008(0.003-0.013,P=0.0030) | 0.016(0.009-0.023,p<0.0001) | 0.002(-0.006-0.010,p=0.649) |
| Too fat vs Too thin | 0.105(0.089-0.122, p<0.0001) | 0.116(0.098-0.135,p<0.0001) | 0.093(0.075-0.112,p<0.0001) |
